# Comprehensive analysis of lactate-related gene profiles and immune characteristics in lupus nephritis

**DOI:** 10.3389/fimmu.2024.1329009

**Published:** 2024-02-22

**Authors:** Zhan Sun, Zhanyan Gao, Mengmeng Xiang, Yang Feng, Jie Wang, Jinhua Xu, Yilun Wang, Jun Liang

**Affiliations:** ^1^ Department of Dermatology, Huashan Hospital, Fudan University, Shanghai, China; ^2^ Department of Dermatology, Huashan Hospital, Fudan University, Shanghai Institute of Dermatology, Shanghai, China

**Keywords:** lupus nephritis, lactate, infiltrating immunocytes, bioinformatics, GEO

## Abstract

**Objectives:**

The most frequent cause of kidney damage in systemic lupus erythematosus (SLE) is lupus nephritis (LN), which is also a significant risk factor for morbidity and mortality. Lactate metabolism and protein lactylation might be related to the development of LN. However, there is still a lack of relative research to prove the hypothesis. Hence, this study was conducted to screen the lactate-related biomarkers for LN and analyze the underlying mechanism.

**Methods:**

To identify differentially expressed genes (DEGs) in the training set (GSE32591, GSE127797), we conducted a differential expression analysis (LN samples versus normal samples). Then, module genes were mined using WGCNA concerning LN. The overlapping of DEGs, critical module genes, and lactate-related genes (LRGs) was used to create the lactate-related differentially expressed genes (LR-DEGs). By using a machine-learning algorithm, ROC, and expression levels, biomarkers were discovered. We also carried out an immune infiltration study based on biomarkers and GSEA.

**Results:**

A sum of 1259 DEGs was obtained between LN and normal groups. Then, 3800 module genes in reference to LN were procured. 19 LR-DEGs were screened out by the intersection of DEGs, key module genes, and LRGs. Moreover, 8 pivotal genes were acquired via two machine-learning algorithms. Subsequently, 3 biomarkers related to lactate metabolism were obtained, including COQ2, COQ4, and NDUFV1. And these three biomarkers were enriched in pathways ‘antigen processing and presentation’ and ‘NOD-like receptor signaling pathway’. We found that Macrophages M0 and T cells regulatory (Tregs) were associated with these three biomarkers as well.

**Conclusion:**

Overall, the results indicated that lactate-related biomarkers COQ2, COQ4, and NDUFV1 were associated with LN, which laid a theoretical foundation for the diagnosis and treatment of LN.

## 1 Introduction

Systemic lupus erythematosus (SLE) is a chronic multifactorial autoimmune disease characterized by multisystemic involvement. Lupus nephritis (LN) is a common type of glomerulonephritis that comprises one of the most major and presenting organ manifestations of SLE, which generally develops early in the course, within the initial 6 to 36 months (1). Predisposing factors contributing to LN include young age, male gender, and non-European lineage (2, 3). The etiological underpinnings of LN encompass abnormalities in B-cell tolerance, production of autoantibodies targeting nuclear and cellular antigens, deposition of immune complexes (ICs) in glomeruli, formation of neutrophil extracellular traps (NETs), and activation of both innate and adaptive immune responses (4). Despite the enhanced understanding of LN pathogenesis, the diagnosis still relies largely on renal biopsy, lacking credible non-invasive biomarkers. Clinically, LN patients commonly present with proteinuria, hematuria, edema, hypertension, and renal insufficiency (5, 6). Although notable strides have been achieved in the management of LN, the remission rate remains unsatisfactory, thereby inevitably leading to end-stage renal disease (ESRD). It is virtually inevitable for lupus individuals to resort to immunosuppressants and glucocorticoids for disease management, which could lead to a spectrum of adverse effects, including infections and cardiovascular involvement (7). Therefore, LN remains a leading cause of morbidity and death among SLE patients. To conclude, timely diagnosis along with prompt and novel therapies are of pivotal importance to LN improvement, which appeals to the identification of new biomarkers.

A continuous emergence of research concerning metabolomics and its correlation with lupus has shed light on novel biomarkers involved in pathogenesis. Notably, the metabolomics studies of lupus patients’ serum or plasma have reported a transparent sluggishness of energy metabolism pathways such as glycolysis and Krebs cycle, as indicated by accumulated glucose but reduced lactate and pyruvate (8, 9). Lactate has long been simply viewed as the by-product of glycolysis, while it is now considered to be a fundamental carbon substrate in cellular metabolism, serving as a signaling molecule in physiological, chronically inflamed, and neoplastic tissue environments as well (10, 11). A novel role attributed to lactate is the protein lactylation, which has recently emerged as a post-translational modification (PTM) of proteins for modulating gene expression (12). Lactate can promote macrophage polarization toward the M2 phenotype via histone lactylation, thereby restraining immune reactions within the tumor microenvironment (TME) (13). Protein lactylation not only reveals a novel realm to the study of protein PTMs but offers a brand-new direction for lactate’s involvement in tumors or autoimmune diseases as well (14). As can be seen, the identification of lactate-related genes (LRGs) in LN patients would provide a new direction for LN diagnosis and treatment.

For this reason, we employed bioinformatics approaches to identify differential LRGs of LN patients from the GEO database and screened important ones through machine-learning methods. We conducted immune infiltration analysis and constructed a transcription network as well, trying to elucidate the principal mechanisms and the relationship between lactate and LN, thus laying clinical significance for the diagnosis and treatment of LN.

## 2 Methods

### 2.1 Data acquisition

**Figure 1:**
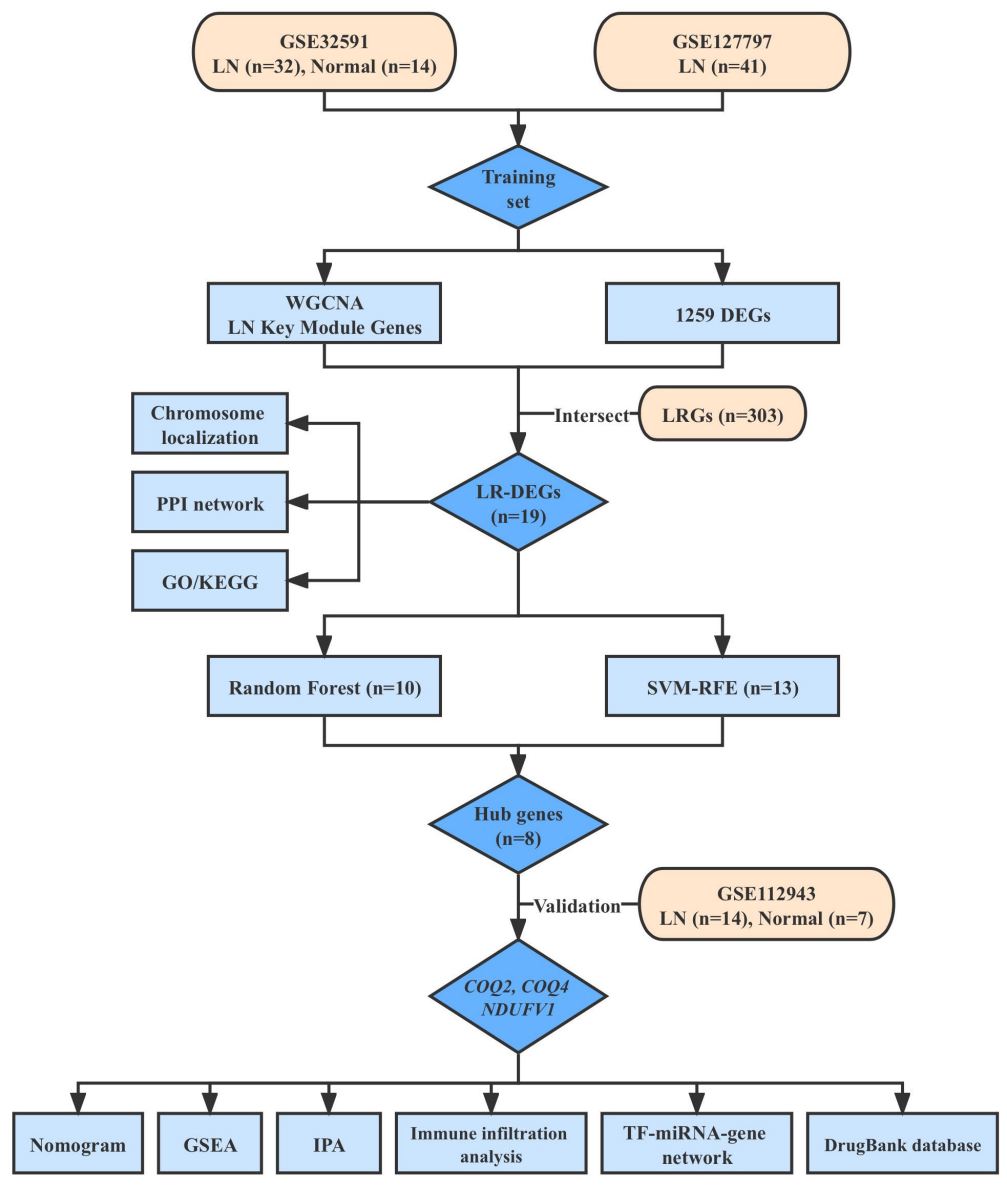
The flow diagram of the research design.

The research flow diagram is presented in **Figure 1**. Three datasets of LN (GSE32591, GSE127797, and GSE112943), which included clinical characteristics and gene expression profiles, were obtained from the Gene Expression Omnibus database (GEO, http://www.ncbi.nlm.nih.gov/geo/). GSE32591 dataset consisted of 14 normal and 32 LN samples of glomeruli (15). GSE127797 dataset included 41 LN samples of glomeruli (16). These two datasets were combined after batch correction using the ‘sva’ to generate a training set (17). GSE112943 dataset, which included 7 normal kidney tissue samples and 14 LN samples, was utilized for further validation (18). A sum of 303 lactate-related genes (LRGs) was obtained from the Molecular Signatures Database (http://www.gsea-msigdb.org/gsea/index.jsp) (19).

### 2.2 Analysis of differentially expressed genes

Principal Component Analysis (PCA) was used to evaluate the availability of the training set via ‘FactoMineR’ and ‘factoextra’ (20). The ‘limma’ package was executed to obtain the differentially expressed genes (DEGs) between normal and LN groups (21). The threshold was set as |log2 fold change (FC)|> 0.5 and adjusted.p.value < 0.05. Volcano plots were applied to show DEGs via the ‘ggplot2’ package. ‘Pheatmap’ was used to create the heatmap for the Top 50 DEGs. To explore the inhibition or activation state of the biological pathway in which DEGs were involved, Ingenuity Pathway Analysis (IPA) was performed (P < 0.05). Z-score > 2 was considered as the activation state and Z-score < −2 was considered as the inhibition state.

### 2.3 WGCNA

The ‘LN’ was considered a clinical trait for WGCNA via the ‘WGCNA’ (version 1.70-3) package in the training set (22). Firstly, we clustered all samples and calculated a hierarchical clustering with the hclust function. The cut-off height was defined as 235 and samples with a height above 235 should be removed as outliers to ensure the accuracy of the analysis. Then, the soft threshold was established, along with trait heatmaps and sample dendrograms. The phylogenetic tree between genes was created after the similarity between genes was determined based on their adjacency. The modules were divided via a dynamic tree-cutting algorithm, and the minModuleSize was 100. Similar modules were combined based on a correlation coefficient of 0.3. Finally, the modules with a certain correlation (|cor| > 0.3, p < 0.05) to LN were used as key modules.

### 2.4 The acquisition of LR-DEGs

The intersection genes of DEGs, key module genes, and LRGs were defined as lactate-related differential expression genes (LR-DEGs) and shown in the Venn diagram. RCircos (1.2.2) was used to plot the chromosome localization circles of LR-DEGs (23). To explore the interaction among LR-DEGs, the Protein-protein interaction (PPI) network was analyzed through the online STRING database (confidence>0.7). GO and KEGG enrichment analysis of LR-DEGs was conducted via the ‘clusterProfiler’ package (24). The p.adjust < 0.05 was selected as significance threshold.

### 2.5 Machine-learning methods

SVM-RFE and random forest (RF) algorithm were applied to screen important genes in the training set (25, 26). The key genes were obtained by pooling the results of these two algorithms. Moreover, using the ‘pROC’ tool, a ROC curve was created to assess the diagnostic utility of the key genes (27). Then, genes with strong diagnostic values for LN (AUC>0.7) and consistent expression trends with the training set and external verification set (GSE112943) were identified as key biomarkers.

### 2.6 Clinical nomogram model

Nomogram has been widely used to predict the probability of individual occurrence of clinical events in clinical research (28). The nomogram containing biomarkers was drawn via ‘rms’ to predict the risk of LN. Evaluation of the predictive effect was done by the calibration and ROC curves.

### 2.7 GSEA and Immune Infiltration Analysis

Single GSEA was conducted to explore the potential KEGG pathways associated with biomarkers through the ‘clusterProfiler’ package (29). The threshold was set as p.adjust < 0.05. In addition, the CIBERSORT algorithm was applied to calculate the relative abundance of 22 immune cells infiltrated in the LN microenvironment (30). Subsequently, Spearman correlation analysis was performed between biomarkers and differential immune cells. The ‘estimate’ package was applied to collect and compare the immunological, stromal, and ESTIMATE scores between normal and LN groups (31). Spearman correlation analysis was performed between biomarkers and these scores via ‘ggExtra’.

### 2.8 Construction of ‘TF-miRNA-gene’ networks

The NetworkAnalyst database was applied to predict the transcription factors (TFs) linked to biomarkers. The miRWalk database was used to predict the miRNAs linked to biomarkers. Moreover, Cytoscape software was applied to optimize the results of the ‘TF-miRNA-gene’ network (32).

## 3 Results

### 3.1 Identification of differentially expressed genes in LN

**Figure 2:**
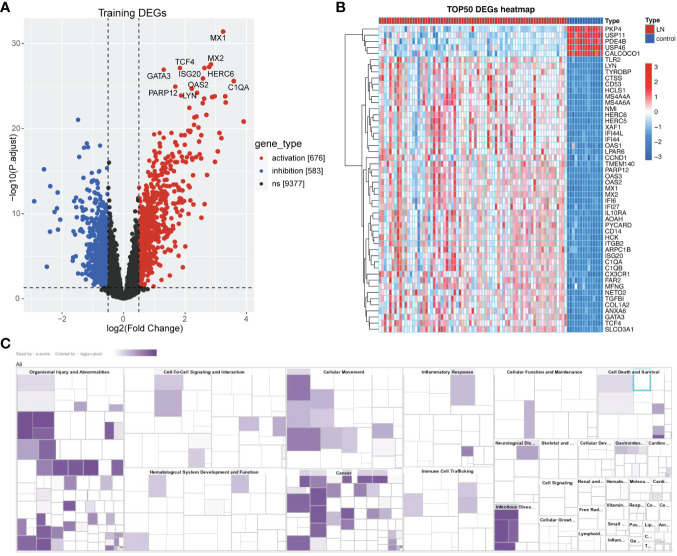
Identification of Lactate-related DEGs in LN. **(A)** Volcano plot showing the DEGs between LN and normal groups, including 583 down-regulated and 676 up-regulated genes. **(B)** Heatmap showing the TOP50 DEGs. **(C)** Disease and function analysis of IPA suggested that these DEGs were associated with ‘cell-to-cell signaling and interaction’ and ‘immune cell trafficking’.

According to PCA results, the merged data set (training set) has eliminated the batch effect (**Supplementary Figure S1A, B**). As shown in **Figures 2A, B**, we obtained the 1259 DEGs between normal and LN groups, including 583 down-regulated and 676 up-regulated genes. In addition, classical pathway analysis of IPA indicated that these DEGs were related to different pathways (**Supplementary Figure S1C**), mainly enriched in ‘pathogen-induced cytokine storm signaling pathway’, ‘phagosome formation’, and ‘LXR/RXR activation’. Disease and function analysis suggested that these DEGs were associated with ‘cell-to-cell signaling and interaction’ and ‘immune cell trafficking’ (**Figure 2C**).

### 3.2 Identification of key module genes associated with LN

**Figure 3:**
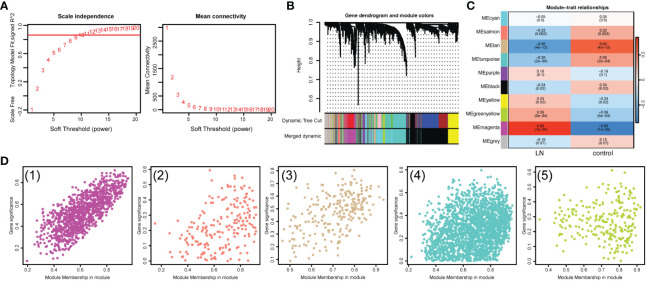
Identification of key module genes associated with LN. **(A)** Analysis of the scale-free fit index and mean connectivity for various soft-thresholding powers (β). **(B)** Gene dendrogram obtained by hierarchical clustering. A total of 10 modules were obtained by the Dynamic Tree Cut algorithm and similar merging. **(C)** Heatmap suggested that the MEturquoise, MEsalmon, MEtan, MEgreenyellow, MEmagenta, and MEblack modules (|cor|>0.3, P<0.05) were markedly correlated with LN. **(D)** Scatterplots of gene significance (GS) versus module membership (MM) showed that 3800 key module genes related to LN were obtained.

To seek out pivotal modules related to LN, WGCNA was conducted. No outlier samples were found, according to the sample clustering results (**Supplementary Figure S2**). The optimal soft threshold was 10. The ordinate scale-free fit index increased when mean connectivity tended to 0, and signed R2 began to approach the critical value of 0.85 (red line) (**Figure 3A**). A total of 10 modules were obtained by the dynamic tree-cut algorithm and similar merging (**Figure 3B**). The MEturquoise, MEsalmon, MEtan, MEgreenyellow, MEmagenta, and MEblack modules (|cor|>0.3, P<0.05) were markedly correlated with LN (**Figure 3C**). Thus, 3800 key module genes related to LN were obtained for subsequent analysis (**Figure 3D**).

### 3.3 Identification of lactate-related DEGs and functional enrichment analysis

**Figure 4:**
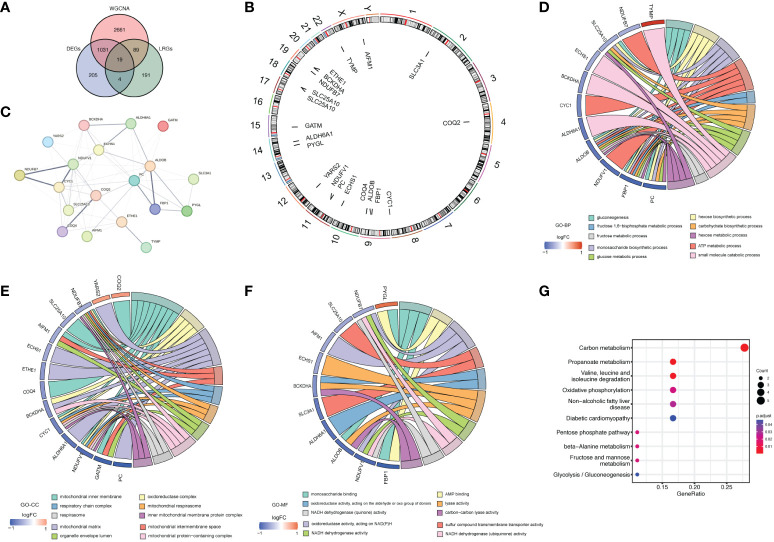
The acquisition and function analysis of LR-DEGs. **(A)** Venn diagram showing 19 LR-DEGs in LN that overlapped DEGs, key module genes, and LRGs. **(B)** Chromosome localization circles of LR-DEGs. **(C)** PPI network of LR-DEGs. **(D–F)** Chord diagrams obtained from the functional enrichment analysis of LR-DEGs. TOP10 GO results indicated that these LR-DEGs were principally involved in the ‘glucose metabolic process’ and ‘small molecule catabolic process’. **(G)** KEGG analysis implied that these LR-DEGs were mainly enriched in the ‘Propanoate metabolism’ and ‘Fructose and mannose metabolism’.

Then, 19 LR-DEGs in LN that overlapped DEGs, key module genes, and LRGs were obtained (**Figure 4A**). All chromosomes, apart from 5, 6, 7, 8, and 16, contained these genes (**Figure 4B**). The PPI network of these LR-DEGs was performed, in which CYC1 and SLC25A10 contained many interdependent proteins. (**Figure 4C**). Functional enrichment analysis was conducted to further probe the function of the LR-DEGs in LN patients. TOP10 GO results indicated that these LR-DEGs were principally involved in the ‘glucose metabolic process’ and ‘small molecule catabolic process’ (**Figure 4D–F**). Additionally, the KEGG analysis implied that these LR-DEGs were mainly enriched in the ‘Propanoate metabolism’ and ‘Fructose and mannose metabolism’ (**Figure 4G**).

### 3.4 Construction and evaluation of the LR-DEGs signature for LN

**Figure 5:**
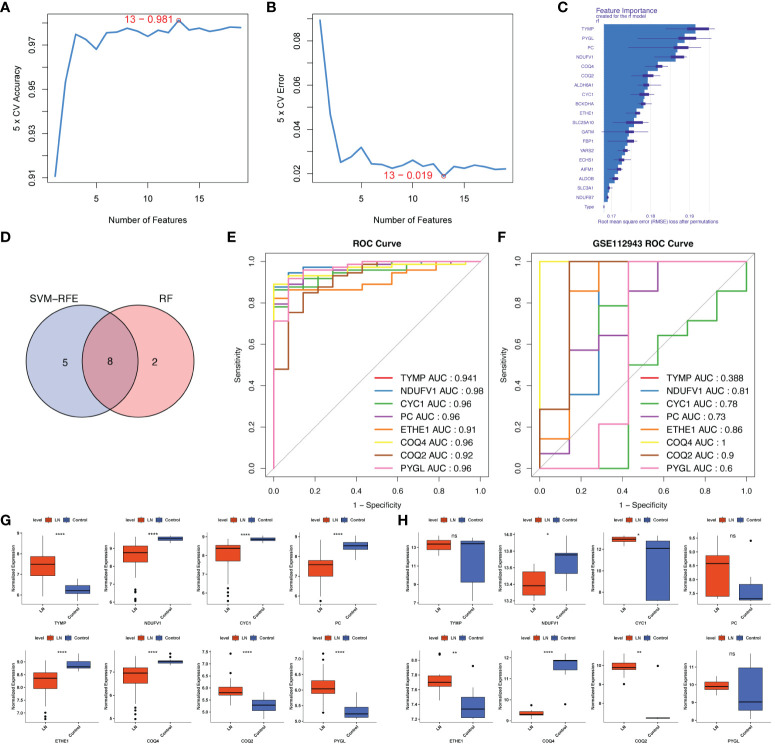
Screening lactated-related biomarkers of LN. **(A, B)** SVM-RFE analysis of 19 LR-DEGs ultimately obtained 13 feature genes. **(C)** RF algorithm showing the TOP10 feature genes. **(D)** Venn diagram identified 8 key LR-DEGs via the intersection of two machine-learning algorithms. **(E, F)** ROC curves of the 8 key genes in the training set and the external validation set. *COQ2*, *COQ4*, and *NDUFV1* demonstrated strong diagnostic values for LN in the external validation set (AUC> 0.7). **(G, H)** The expression pattern of *COQ2*, *COQ4*, and *NDUFV1* in the external validation set was entirely consistent with the training set. ns, not significant. *p < 0.05, **p < 0.01, ****P < 0.0001.

To further dig out the key genes, SVM-RFE analysis was performed on 19 LR-DEGs to unearth the optima. Ultimately, 13 feature genes were obtained, including *TYMP*, *NDUFV1*, *AIFM1*, *CYC1*, *FBP1*, *PC*, *ETHE1*, *COQ4*, *YARS2*, *COQ2*, *PYGL*, *SLC3A1* and *SLC25A10* (**Figures 5A, B**). Meanwhile, the Top10 feature genes were retained by the RF algorithm, including *ETHE1*, *BCKDHA*, *CYC1*, *ALDH6A1*, *COQ2*, *COQ4*, *NDUFV1*, *PC*, *PYGL* and *TYMP* (**Figure 5C**). Correspondingly, we further identified 8 key genes by the intersection of two algorithms, including *TYMP*, *NDUFV1*, *CYC1*, *PC*, *ETHE1*, *COQ4*, *COQ2* and *PYGL* (**Figure 5D**). *COQ2*, *COQ4*, and *NDUFV1* demonstrated strong diagnostic value for LN in the external validation set (AUC> 0.7). The expression pattern was entirely consistent with the training set. These three genes were discovered to be lactate-related biomarkers (**Figures 5E–H**).

### 3.5 Clinical and functional enrichment analysis of biomarkers with LN

**Figure 6:**
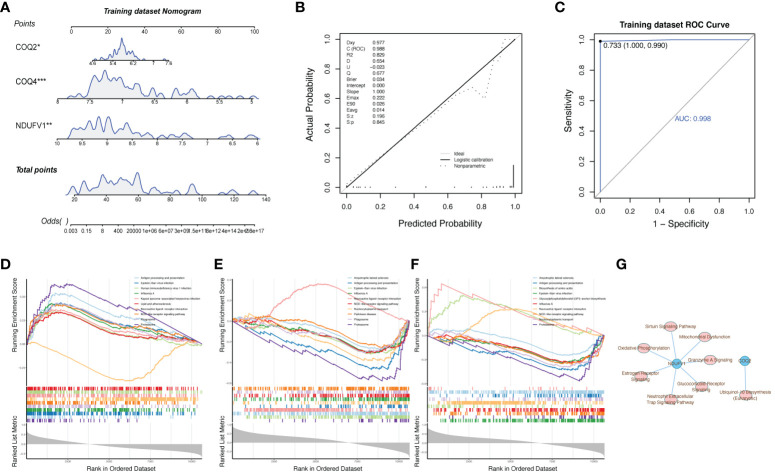
Clinical and functional enrichment analysis of biomarkers with LN. **(A)** Nomogram containing biomarkers. **(B, C)** Calibration and ROC curves proved the feasibility of the nomogram. **(D–F)** Single-gene GSEA of *COQ2*, *COQ4*, and *NDUFV1* showed that these three biomarkers were related to ‘antigen processing and presentation’ and ‘NOD-like receptor signaling pathway’. **(G)** Classical pathway analysis of IPA.

To further explore the relationship between biomarkers and LN, the nomogram containing biomarkers was generated (**Figure 6A**). The calibration and ROC curves proved that the feasibility of the nomogram was effective (**Figures 6B, C** **)**. To further study the potential roles of *COQ2*, *COQ4*, and *NDUFV1* in LN, we performed single-gene GSEA on these 3 biomarkers. The KEGG results showed that these three biomarkers were related to ‘antigen processing and presentation’ and ‘NOD-like receptor signaling pathway’ (**Figures 6D–F**). The classical pathway analysis of IPA indicated that *NDUFV1* was related to ‘Oxidative Phosphorylation’ and ‘mitochondrial dysfunction’, while *COQ2* was related to ‘Ubiquinol-10 Biosynthesis’ (**Figure 6G**).

### 3.6 The role of *COQ2*, *COQ4*, and *NDUFV1* in LN immune microenvironment

**Figure 7:**
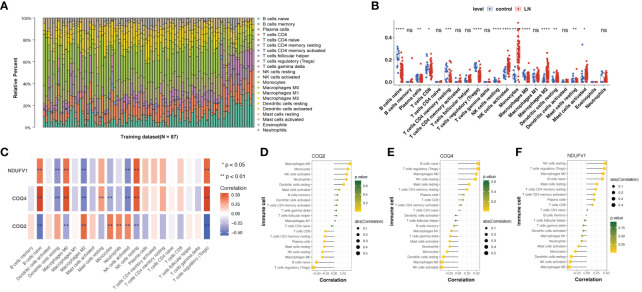
Immune infiltration analysis of lactate-related biomarkers in LN. **(A)** Relative proportions of immune infiltration in LN. **(B)** Abundances of 13 immune cells differed significantly in LN. **(C–F)** Correlation analysis of biomarkers and twenty-one kinds of immune cells showed that Macrophages M0 and T cells regulatory (Tregs) were positively associated with *COQ4* and *NDUFV1*; they were negatively associated with *COQ2*. ns, not significant. *p < 0.05, **p < 0.01, ***p < 0.001, ****P < 0.0001.

Since the pathophysiology of LN patients and the immune microenvironment were related, the immune microenvironment in LN was further explored. The expression abundance of 22 types of immune cells was analyzed (**Figure 7A**). Notably, 13 immune cell abundances differed significantly in LN samples, including Tregs, Macrophages M0 and M2, Monocytes, naïve B cells, CD8+ T cells, CD4+ memory resting T cells, both resting and activated NK and Mast cells, Plasma cells and resting Dendritic cells (DCs) (**Figure 7B**). Then, we analyzed the correlation between biomarkers and differential immune cells, finding that Macrophages M0 and T cells regulatory (Tregs) were positively associated with *COQ4* and *NDUFV1*; they were negatively associated with *COQ2* (**Figures 7C–F**).

**Figure 8:**
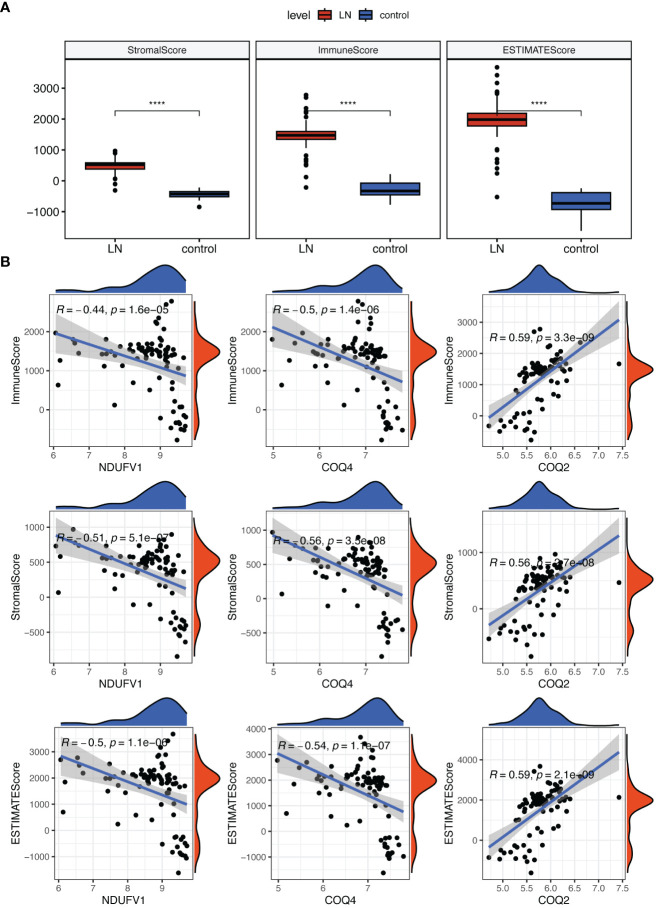
Analysis of the role of biomarkers in LN immune microenvironment. **(A)** The Stromal, Immunological, and ESTIMATE scores were all higher in LN compared to the control group. **(B)** Correlation analysis indicated that these three scores were negatively correlated with *COQ4* and *NDUFV1*, but positively correlated with *COQ2*. ****P < 0.0001.

In addition, we discovered that the LN group had higher stromal, immunological, and estimate scores (**Figure 8A**). These three scores were negatively correlated with *COQ4* and *NDUFV1* but positively correlated with *COQ2* (**Figure 8B**).

### 3.7 Analysis of regulatory network and drug in LN

**Figure 9:**
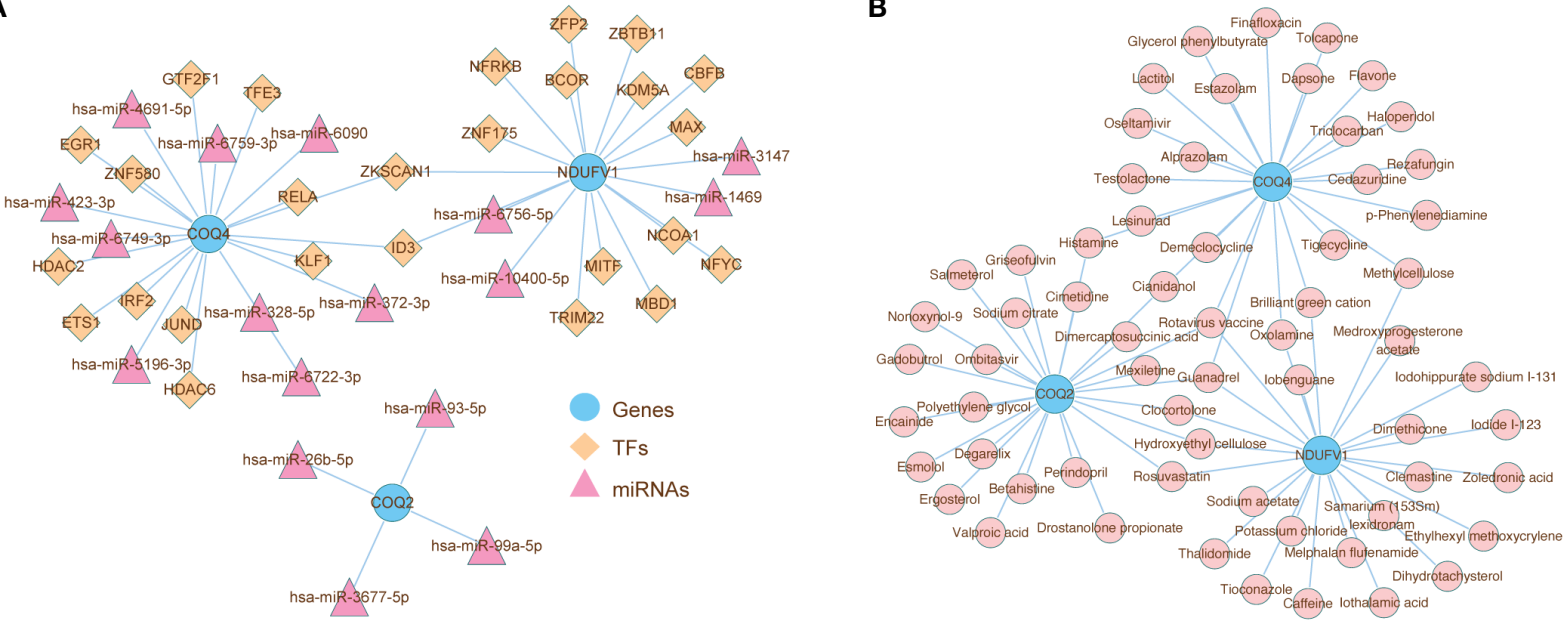
Analysis of the role of biomarkers in LN immune microenvironment. **(A)** ‘TF-miRNA-gene’ network presenting the regulatory mechanisms of *COQ2*, *COQ4*, and *NDUFV1*, which had 46 nodes and 45 edges. **(B)** The relationship between biomarkers and drugs predicted from the DrugBank database.

The ‘TF-miRNA-gene’ network was created to investigate the regulatory mechanisms of *COQ2*, *COQ4*, and *NDUFV1*, which had 46 nodes and 45 edges (**Figure 9A**). In the network, *ZKSCAN1* might simultaneously affect the expression of *COQ4* and *NDUFV1*. The hsa-miR-93-5p regulated the expression of *COQ2*. Drugs that targeted *COQ2*, *COQ4*, and *NDUFV1* were predicted in the DrugBank database. The relationship between biomarkers and drugs was shown in **Figure 9B**, including 63 nodes and 75 edges. Both the Rotavirus vaccine and Guanadrel were found to be co-targeted drugs with three biomarkers, which might play an important role in the treatment of LN.

## 4 Discussion

SLE is an autoimmune disease of unknown etiology, characterized by multi-system damage and the formation of multiple autoantibodies against nuclear, cytoplasmic, and membranous antigens. With a subtle or sudden onset, nearly 70 percent of SLE patients would develop LN and suffer from a series of symptoms of glomerular, tubulointerstitial, and renal vascular damage (33). Any SLE patient with renal lesions should pay attention to whether LN is accompanied, and renal biopsy should be performed for pathological examination. Despite the enhanced understanding of LN, a lack of non-invasive diagnostic biomarkers and essential treatment alternatives remains a major obstacle to the prognosis of LN. The metabolic disturbances that underlie autoimmune diseases have been studied recently. Lactate-mediated signaling pathways have turned out to conduce to both cancer progression and inflammatory diseases (34). Lactate has long been recognized as the end product of glycolysis and viewed as simply a waste product. However, in both TME and the inflammatory disease microenvironment, lactate, produced by infiltrating immune cells through glycolysis, triggers a series of intra- and extracellular signals, contributing to both tumor progression and constant inflammation (35, 36). Integrated profiling of the SLE metabolome has manifested heightened inflammation, oxidative stress, and reduced energy generation (37), as indicated by accumulated glucose but reduced lactate, while the mechanisms remain unknown. We believe that the identification of lactate-related molecules in LN would contribute to both the diagnosis and treatment of LN pathogenesis. Moreover, since LN is characterized by the failure to maintain immune tolerance (38), immune infiltration studies of LN and lactate-related biomarkers would further explain the role of immune cells in LN pathogenesis.

Our study is based primarily on the transcriptome data of LN patients from GEO database and the 303 known lactate-related genes downloaded from the MsigDb database. Bioinformatics analysis was carried out to explore the underlying connections between LN and lactate metabolism. The results indicated that lactate-related biomarkers *COQ2*, *COQ4*, and *NDUFV1* were associated with LN. *COQ2* and *COQ4* are components of an enzyme complex involved in the biosynthesis of coenzyme Q10 (CoQ10) (39, 40), which is a mitochondrial electron carrier responsible for generating adenosine triphosphate (ATP) and an essential lipophilic antioxidant located in plasma lipoproteins and membranes (41). Mutations in the *COQ2* and *COQ4* genes can lead to CoQ deficiency disorders such as multiple system atrophy and primary coenzyme Q10 deficiency (42, 43), which often result in mitochondrial dysfunction and a wide range of symptoms, including muscle weakness, neurologic abnormalities, and cardiomyopathy (44). Mitochondrial dysfunction and metabolic disturbances have been identified as key regulators of lupus and other autoimmune diseases. Research has found that idebenone, which is a synthetic quinone analog compound of CoQ10, could modify survival in murine lupus through the regulation of mitochondrial functions and improve immune dysregulation and organ damage. MRL/*lpr* mice fed with idebenone displayed lower serum creatinine concentration and reduced the severity of nephritis through histologic analysis of kidneys (45, 46). Taken together, it is not hard to notice that new agents modulating oxidative stress and mitochondrial metabolism might have a potent therapeutic role in the treatment of LN; Complex I (CI, NADH: ubiquinone oxidoreductase) refers to mitochondrial oxidative phosphorylation (OxPhos) enzyme complex consisting of 45 subunits, and its dysfunction would generally impair energy production and affect various organs including kidneys (47). NADH: Ubiquinone Oxidoreductase Core Subunit V1 (NDUFV1) is a nuclear-encoded structural subunit of CI and its mutations are associated with Leigh syndrome (LS), diffuse leukoencephalopathy, and Parkinson’s disease (48, 49). According to previous research, the reinforcement expression of *NDUFV1* has turned out to reduce serum creatinine and blood urea nitrogen, attenuate proximal tubule damage, and repress cell apoptosis in renal ischemia/reperfusion (I/R) mice, which may be due to the improved mitochondrial metabolism, and reduced oxidative stress by overexpressed *NDUFV1* (50, 51). Having shown that the expression level of *NDUFV1* was decreased in LN in our study and the classical pathway analysis of IPA indicated that *NDUFV1* was related to ‘oxidative phosphorylation’ and ‘mitochondrial dysfunction’, we thus speculate that *NDUFV1* deficiency could impair mitochondrial metabolism and homeostasis in renal tissue, contributing to the progress of LN. Therefore, targeting *NDUFV1*, a representative of mitochondrial Complex I, should be a promising strategy for treating renal impairment in LN.

Furthermore, we developed the nomogram containing these 3 biomarkers to predict LN and the calibration and ROC curves proved its feasibility, which indicated that lactate-related genes might work as novel non-invasive biomarkers for LN diagnosis. To understand the potential roles of *COQ2*, *COQ4*, and *NDUFV1* in LN, we performed single-gene GSEA and the KEGG results indicated that these three biomarkers were related to ‘antigen processing and presentation’ and ‘NOD-like receptor signaling pathway’. Based on the dissection of cell-extrinsic suppressive pathways, it has been established that lactic acid in TME inhibits type-I interferon (IFN) downstream of Toll-like receptor 3 (TLR3) and the cytosolic sensors STING. As a result of DC conditioning by lactate, antigen degradation was accelerated, and cross-presentation was impaired (52). The activation of type-I IFN is one of the most important factors contributing to lupus pathogenesis (53). Therefore, we reasonably speculate that sluggishness of lactate levels in LN may impair the inhibition of type-I IFN production through the pathways mentioned above, which in turn leads to the progress of the disease. At the same time, it remains unclear whether targeting LRGs, such as *COQ2*, *COQ4*, and *NDUFV1*, could simulate the conditions of TME, in the microenvironments of inflammatory diseases and thus significantly reprogram DC-mediated innate immune responses and antigen processing; Consistent with our enrichment results, NOD-like receptor signaling pathway is affected by lactate levels in previous research. The intracellular receptor NOD-like receptor protein 3 (NLRP3) possesses the capability to distinguish extrinsic pathogens and endogenous danger signals. It assembles the adaptor ASC and caspase-1 to form an oligomeric complex called the NLRP3 inflammasome (54). Lactate could mitigate the activation of NLRP3 inflammasome, which in turn impacts the production of pro-inflammatory cytokines via G protein-coupled receptor 81 (GPR81) signaling (55). Research has found that inhibition of NLRP3 inflammasome via receptor-interacting protein kinase 3 (RIP3) would lead to the amelioration of LN and the decline of auto-antibody production (56), which suggests that various signaling pathways for the activation of NLRP3 inflammasome are operative in the pathogenesis of lupus and targeting LRGs might work with LN via NLRP3.

The immune infiltration studies showed differences in multiple immune cells between LN and control groups, and Tregs were found to be positively associated with *COQ4* and *NDUFV1*, while negatively associated with *COQ2*. Tregs serve a crucial function in maintaining immune homeostasis, which could target T cells via regulating antigen-presenting cells by expressing anti-inflammatory cytokines such as IL-10 and TGF-β (57). B cells producing autoantibodies, which contributed to lupus progress, could be suppressed by Tregs directly as well (58). In individuals with lupus, the amount of Tregs and their ability to inhibit the proliferation of effector T cells are greatly reduced, while the proportion of effector T cells is significantly increased. This imbalance results in a breakdown of immune tolerance to self-antigens, causing damage to multiple tissues and organs (59). However, in TME, the ability of Tregs to differentiate, proliferate, and suppress the anti-tumor immune system would be enhanced by lactate, via the expression of *FOXP3* (60). Researchers have highlighted the antagonistic effect of glucose on Treg function and properties, while lactate can both be used by Tregs as fuel and protect their high suppressive capacity from the negative effects of glucose as well (61).

Meanwhile, local treatment with lactate has been found to effectively prevent intestinal inflammation and histopathological damage in the colitis model (62). Other researchers have found that through a hypoxia-inducible factor 1α (HIF-1α) mediated mechanism, lactate from immune cells like activated DCs could facilitate the expression of NDUFA4L2. NDUFA4L2 then limited mitochondrial reactive oxygen species, leading to the activation of XBP1-driven transcriptional modules in DCs, which could control pathogenic autoimmune T cells. Based on this, they developed a synthetic lactate-producing probiotic, which successfully suppressed T cell-driven central nervous system autoimmunity in experimental autoimmune encephalomyelitis (EAE) models through the activation of HIF-1α–NDUFA4L2 signaling in DCs (63). Collectively, we formulate the hypothesis that lactate treatment or targeting lactate-related biomarkers might be a novel treatment strategy for LN, while the underlying mechanisms of lactate in autoimmune diseases like LN need further research.

To our knowledge, this is the first bioinformatic research to explore the relationship between LN and lactate-related genes. However, there are still several limitations to this study. First, we have not verified the differential expression of LRGs in kidney samples of LN patients and normal controls due to the difficulties in sample collection. According to current academic research, single-cell analysis of both the peripheral blood mononuclear cells (PBMCs) (64) and the kidney biopsies (65) of lupus nephritis has shown the immune cell landscape of the disease. Further analysis of the datasets from these articles could show the expression profiles of the hub LRGs in various immune cells of LN and validate our main findings. However, the validation analysis was hard to accomplish due to the data acquisition issues. Second, the lack of experimental data makes it hard to explain the underlying mechanisms. Third, we didn’t differentiate between various pathological types of LN, such as proliferative and membranous lupus nephritis. Despite that future investigations are necessary to validate our conclusions, this study still sheds light on novel biomarkers involved in LN pathogenesis.

## 5 Conclusion

In conclusion, this study has identified 3 lactate-related hub genes that demonstrated strong diagnostic value for LN, including *COQ2*, *COQ4*, and *NDUFV1* They might contribute to LN pathogenesis via ‘antigen processing and presentation’ and ‘NOD-like receptor signaling pathway’. Macrophages M0 and Tregs were also associated with these 3 biomarkers. This study provides valuable insights for elucidating the lactate’s role in LN and the 3 biomarkers could lay a theoretical foundation for the diagnosis and treatment of LN.

## Data availability statement

The datasets for this study can be found in online repositories. The names of the repository/repositories and accession number(s) can be found in the article/**Supplementary Material**.

## Author contributions

ZS: Conceptualization, Formal analysis, Methodology, Visualization, Writing – original draft. ZG: Writing – review & editing. MX: Writing – review & editing. YF: Writing – review & editing. JW: Writing – review & editing. JX: Writing – review & editing. YW: Writing – review & editing. JL: Writing – review & editing.
