# Supplementary material for: Comprehensive analysis of lactate-related gene profiles and immune characteristics in lupus nephritis
Source: Front Immunol. 2024 Feb 22;15:1329009. doi: 10.3389/fimmu.2024.1329009 (PMC10917958; doi:10.3389/fimmu.2024.1329009)
Supplement: Supplementary file 5 [file DataSheet_5.zip › Supplementary Figures/Supplementary_FIgure_Legends.docx]

Supplementary Material


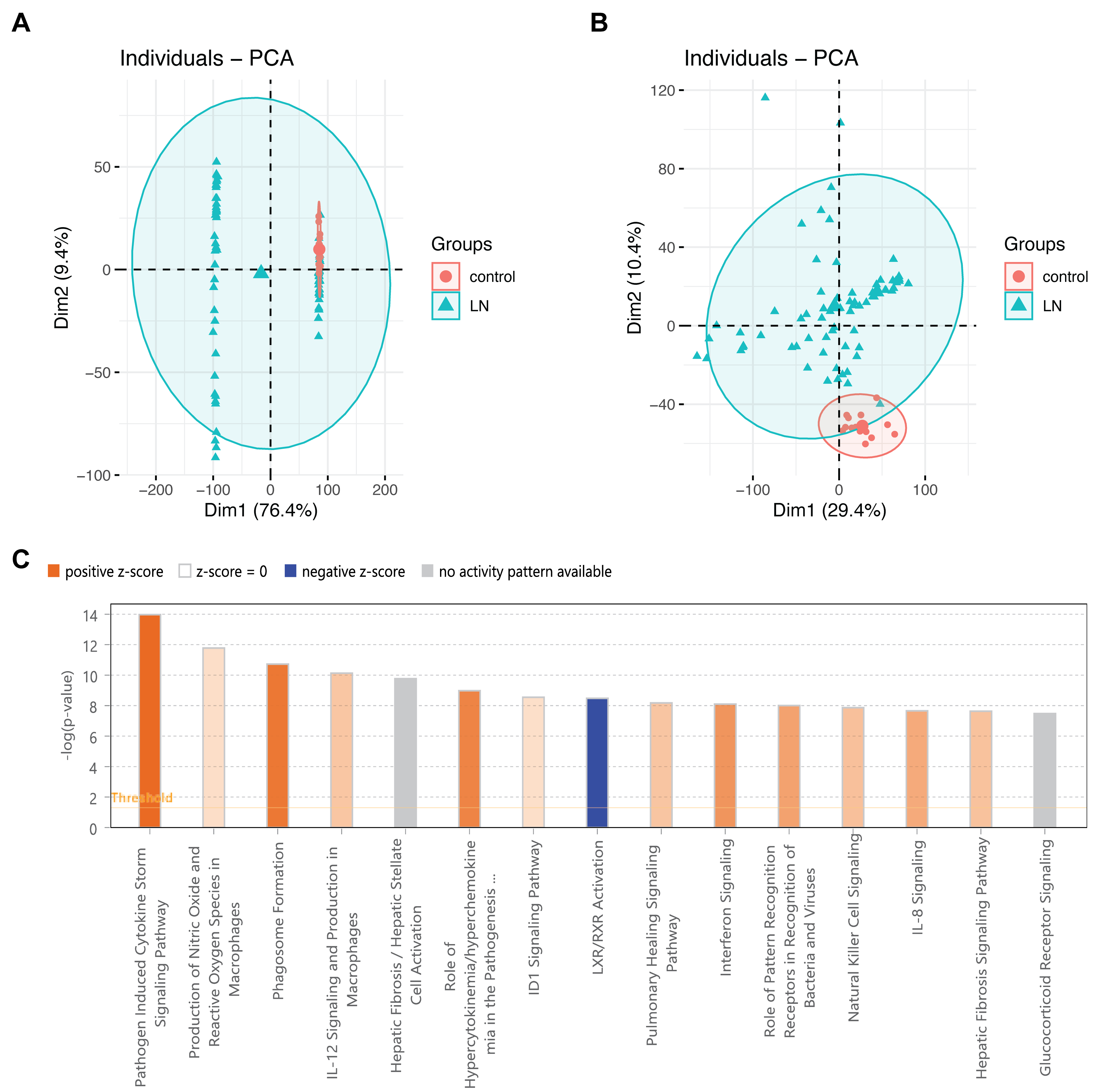


**Supplementary Figure 1.** The merged data set (training set) has eliminated the batch effect. **(A-B)** Principal component analysis shows the date of the training set before and after the batch correction. **(C)** The classical pathway analysis of IPA indicated pathways these DEGs related to. The ‘pathogen-induced cytokine storm signaling pathway’ and ‘phagosome formation’ were activated, while ‘LXR/RXR activation’ was inhibited.


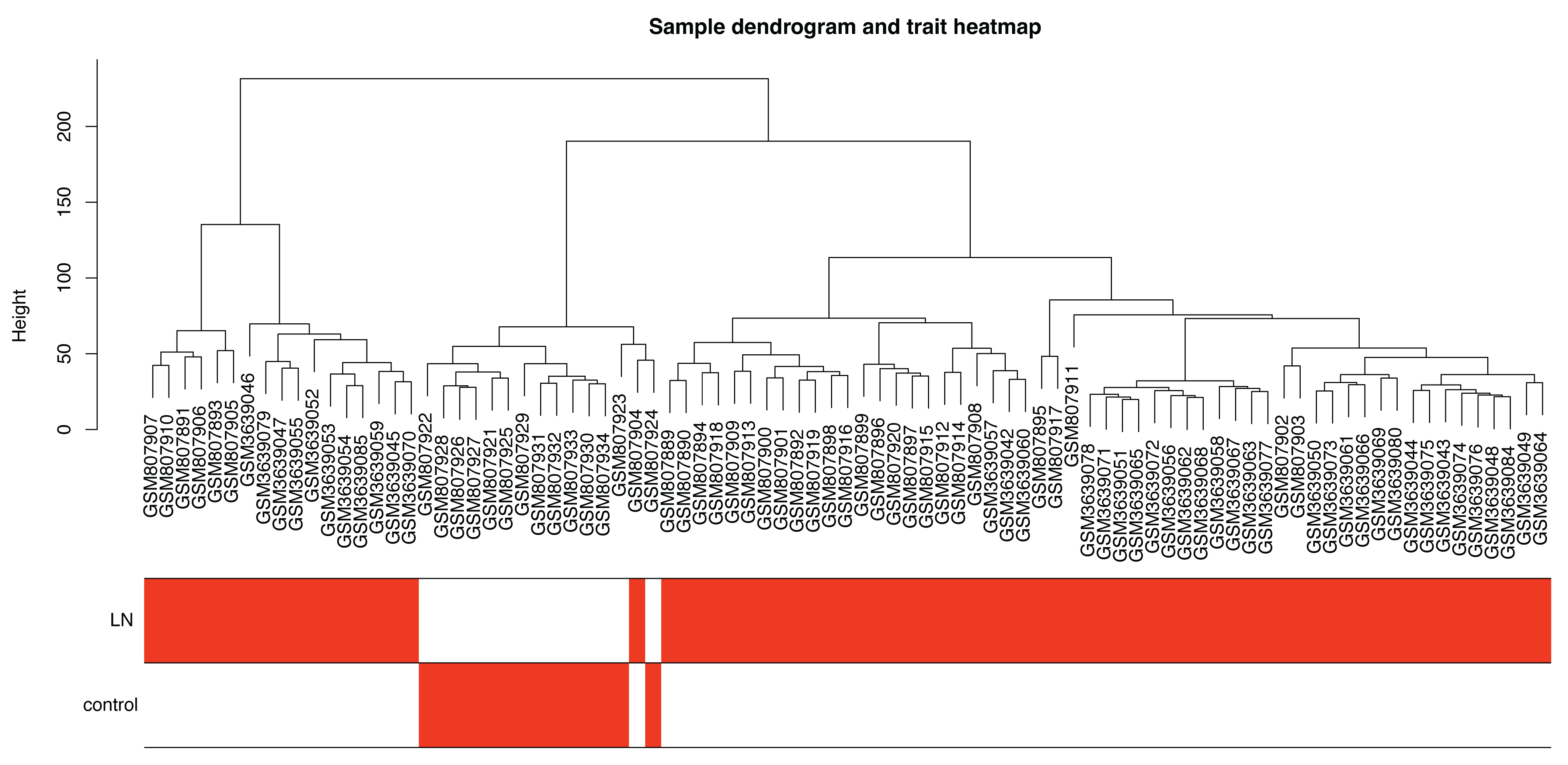


**Supplementary Figure 2.** Sample dendrogram and trait heatmap. According to the sample clustering results, no outlier samples were found.
