# Supplementary figures and images for: Comprehensive analysis of lactate-related gene profiles and immune characteristics in lupus nephritis
Source: Front Immunol. 2024 Feb 22;15:1329009. doi: 10.3389/fimmu.2024.1329009 (PMC10917958; doi:10.3389/fimmu.2024.1329009)

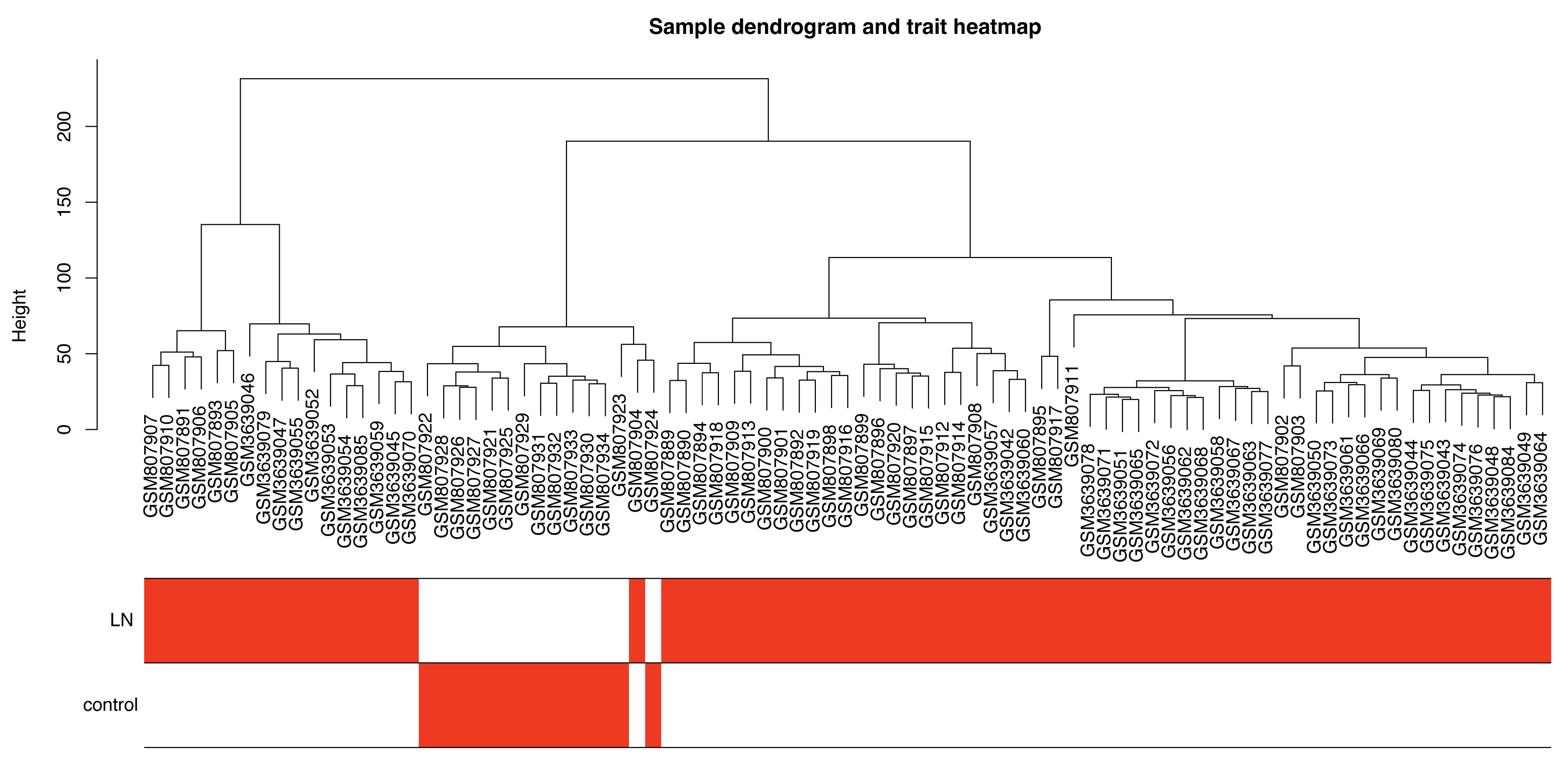

Supplement: Supplementary file 5 [file DataSheet_5.zip › Supplementary Figures/Fig_S2.tif]

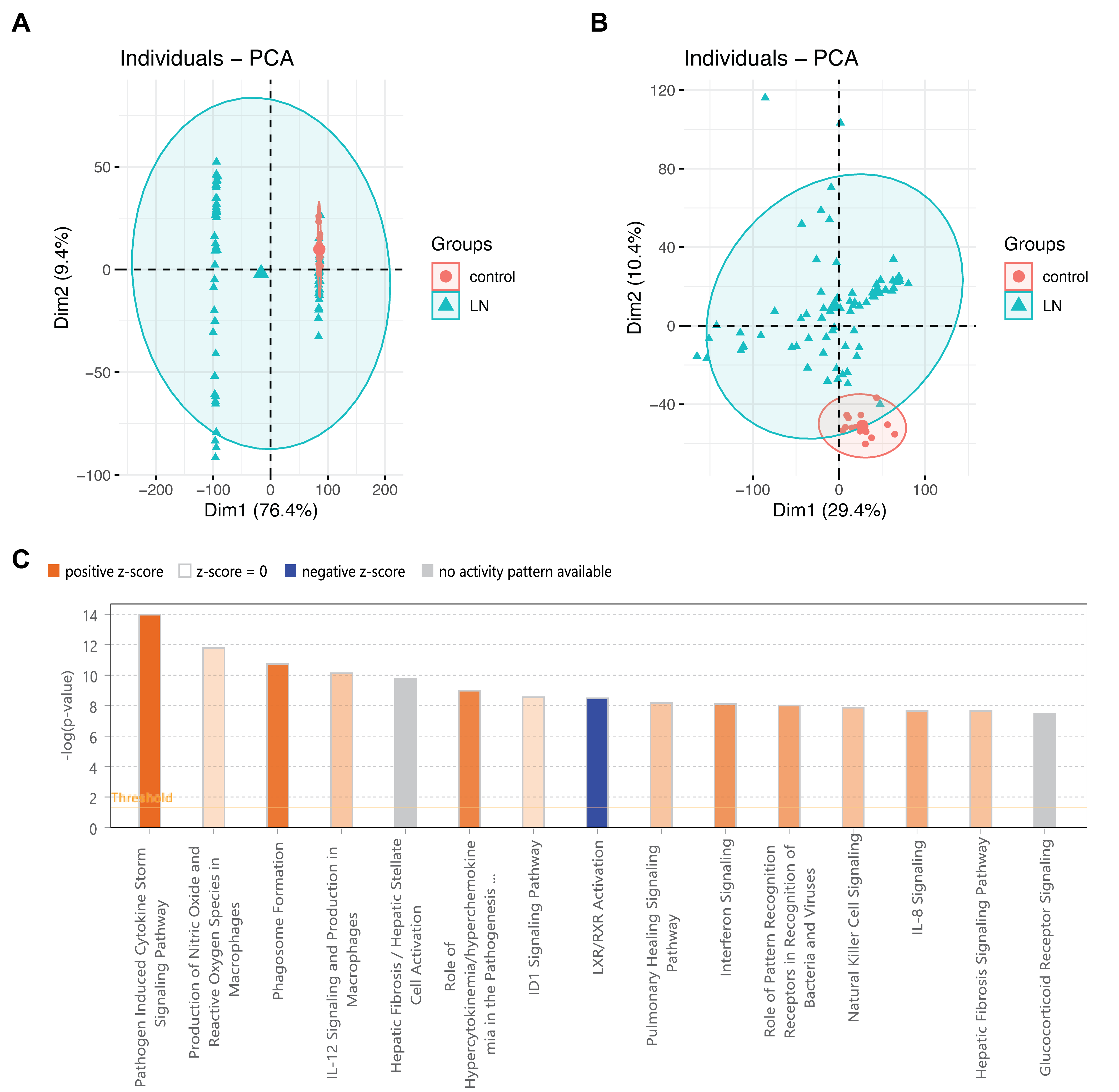

Supplement: Supplementary file 5 [file DataSheet_5.zip › Supplementary Figures/Fig_S1.tif]
